# Modulation of the Tumour Microenvironment by HER2 in Oesophagogastric Adenocarcinoma: Implications for Tumour Progression, Therapeutic Resistance, and Clinicopathological Outcomes

**DOI:** 10.3390/cancers17243987

**Published:** 2025-12-14

**Authors:** Nicola B. Raftery, Mark Ward, Narayanasamy Ravi, John V. Reynolds, Jessie A. Elliott, Claire L. Donohoe

**Affiliations:** 1 Department of Upper GI Surgery, National Centre for Esophageal and Gastric Cancer, Trinity St. James’s Cancer Institute, St James’s Hospital, D08 NHY1 Dublin, Ireland; 2 Department of Pharmacology and Therapeutics, Trinity St. James’s Cancer Institute, St James’s Hospital, D08 NHY1 Dublin, Ireland

**Keywords:** oesophagogastric cancer, HER2 amplification/overexpression, tumour microenvironment, drug resistance, immunotherapy

## Abstract

HER2 (human epidermal growth factor receptor 2) is a receptor tyrosine kinase which is overexpressed in ~20% of patients with oesophagogastric adenocarcinoma (EGA). HER2 represents a targetable transmembrane glycoprotein receptor of the epidermal growth factor receptor (EGFR) family, which plays a crucial role in cell proliferation, survival, and differentiation. HER2 significantly influences the tumour microenvironment (TME) through various mechanisms, creating a niche that supports tumour progression, immune evasion, and therapeutic resistance. In HER2-positive EGA, aberrant signalling pathways, such as PI3K/AKT and MAPK/ERK, enhance tumour cell survival and proliferation, whilst upregulation of angiogenic factors like VEGF fosters vascularization, meeting a tumour’s metabolic demands and facilitating its proliferation. HER2 also modulates the tumour immune microenvironment (TIME) by downregulating MHC molecules and recruiting immunosuppressive cells, including regulatory T-cells (T-reg) and tumour-associated macrophages (TAMs), which release cytokines that further inhibit anti-tumour immune responses. Together, these factors foster a pro-inflammatory, immunosuppressive microenvironment that underpins resistance to HER2-targeted therapies. As more HER2-directed treatments become available, such as trastuzumab–deruxtecan (T-DXd), gaining a deeper understanding of the multifaceted influence of HER2 on the TME in EGA will be crucial for the development of improved targeted treatments that can overcome these challenges and lead to advancements in targeted treatment for HER2-overexpressing EGA. This review provides a comprehensive overview of the impact of HER2 on the TME in EGA and highlights the challenge it represents as well as the opportunity for novel therapeutic development and the implications for patients in terms of clinicopathological outcomes.

## Simple Summary

Oesophagogastric cancers are often aggressive and difficult to treat, especially when they produce high levels of a protein called HER2. This protein helps cancer cells grow and spread, but it also changes the environment around a tumour (microenvironment). Understanding how HER2 shapes the tumour microenvironment is important, because it can explain why some patients do not respond well to current therapies. New drugs targeting HER2 are now being developed, and their success may depend on overcoming these resistance mechanisms. In this review, we explore how HER2 affects the tumour microenvironment, why this makes treatment challenging, and how future research could lead to better therapies that could improve the survival and quality of life for patients with HER2-positive oesophagogastric cancer.

## 1. Introduction

Worldwide, oesophagogastric adenocarcinoma (EGA) ranks among the most prevalent and lethal malignancies, with roughly 1.6 million new diagnoses and more than 700,000 deaths each year [1,2,3]. Even with the progress in multimodal treatment strategies over the past two decades, the median overall survival (OS) remains poor, with a five-year survival rate of only about 25% [3,4]. There is an urgent need for targeted, effective treatments for patients with EGA to address the dismal prognosis associated with the condition.

HER2 is a receptor tyrosine-protein kinase that is encoded by the ERBB2 gene on chromosome 17 and plays a key role in tumour biology [5]. It is composed of three major regions: an extracellular domain, a single transmembrane domain, and an intracellular tyrosine kinase domain [6]. Unlike other receptors in the same family, HER2 does not have a known ligand. Instead, its activation occurs through dimerization, which initiates intracellular signalling cascades that regulate processes such as cell proliferation, apoptosis, adhesion, migration, and differentiation [6,7]. In carcinomas, HER2 functions as an oncogene, with gene amplification leading to protein overexpression at the cell membrane. This overexpression drives oncogenic signalling and contributes to the malignant transformation and progression of tumour cells [8]. HER2 amplification refers to increased copies of the ERBB2 gene, whereas overexpression describes elevated HER2 protein levels, and while amplification often drives overexpression, the two do not always perfectly correlate [9].

Approximately 20% of EGA tumours exhibit HER2 overexpression or amplification [10]. HER2 expression in EGA is markedly more heterogeneous than in breast cancer, with patchy, incomplete, or regionally variable amplification across a tumour. This heterogeneity not only complicates accurate assessment and therapeutic targeting, but also contributes to differential sensitivity to HER2-directed therapies and uneven modulation of the local immune microenvironment [11]. For patients with advanced or metastatic disease, HER2 positivity is a key biomarker, identifying those who may benefit from trastuzumab in combination with chemotherapy, which has been shown to improve OS, and testing for HER2 is standard of care for this patient cohort [12]. More recently, the results from KEYNOTE-811 established that adding the immune checkpoint inhibitor (ICI) pembrolizumab to trastuzumab and chemotherapy significantly improves the survival outcomes in first-line HER2-positive metastatic gastric and junctional tumours. The interim analysis led to the regulatory approval of trastuzumab in this context, and the final analysis of OS supported this rationale and were presented at ESMO 2024 [13,14]. In contrast, the role of HER2-directed treatment in patients undergoing curative-intent treatment (e.g., perioperative chemotherapy or surgery) is less well defined and HER2 status is not yet routinely used to guide therapy in the curative-intent cohort.

HER2-driven signalling has been shown to remodel the TME through multiple mechanisms. The activation of angiogenic programs, including upregulation of vascular endothelial growth factor (VEGF), facilitates angiogenesis, supporting rapid growth and nutrient delivery [15]. Concurrently, HER2 signalling contributes to immune evasion by downregulating antigen presentation mechanisms, including MHC class I molecules, and by recruiting immunosuppressive cell populations, such as T-reg cells and TAMs [16,17,18]. These immune cells secrete cytokines and growth factors that reinforce a pro-inflammatory yet immunosuppressive milieu, facilitating tumour progression and reducing the efficacy of immune-mediated tumour clearance [19]. Collectively, these HER2-driven changes establish a TME that is permissive of tumour growth and resistant to targeted therapy.

This review aims to provide a comprehensive overview of the impact of HER2 on the TME in EGA, summarize the mechanisms by which HER2 signalling can shape tumour progression and immune evasion, and explore the implications of these findings for HER2-directed treatments. By integrating insights from preclinical and clinical studies, we highlight both the challenges and opportunities that HER2 represents as a target for future combination therapy approaches and personalized treatment strategies.

## 2. HER2: Biology and Mechanisms of Action

### 2.1. HER2 Signalling Pathways

**Figure 1:**
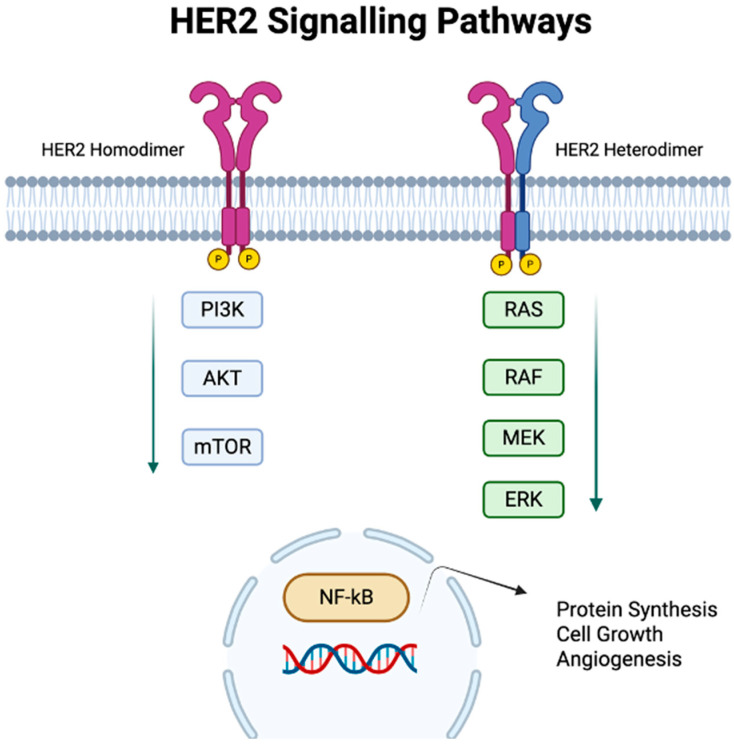
HER2 signalling pathways. HER2 dimerization at the cell membrane triggers multiple oncogenic processes: (1) activation of PI3K/AKT/mTOR and (2) RAS/RAF/MEK/ERK signalling pathways, with downstream activation of NFκb resulting in stimulation of protein synthesis, cell growth, and angiogenesis.

The HER2 receptor has no known ligand that directly binds to it; instead, it is capable of forming both homodimers and heterodimers with other members of the EGFR family in a ligand-independent fashion [19]. This dimerization is crucial for initiating its downstream signalling pathways (Figure 1). The dimerization process can vary across different cancer types. EGA is a very heterogenous cancer type, with HER2 expression being variable within a tumour. Although HER2 homodimers generate relatively modest downstream signalling, heterodimers, particularly HER2/HER3 complexes, are among the most potent activators of mitogenic and survival pathways. HER3 is a key partner in this process, as its multiple binding sites for the PI3K p85 regulatory subunit enable strong and sustained activation of the PI3K/AKT cascade when paired with HER2 [19,20]. Activation of the PI3K/AKT pathway promotes cell survival and growth and can lead to the downstream activation of mTOR (mammalian target of rapamycin), which, in turn, leads to protein synthesis and cell growth [21]. Parallel activation of the Ras/RAF/MEK/ERK cascade further promotes tumorigenesis through ERK’s activation of both the mTOR and JNK pathways, modulating key cellular processes, including proliferation and apoptosis [19,22]. Activation of both the PI3K/AKT and Ras/RAF/MEK/ERK pathways by HER2 can also lead to upregulation of HER2 transcription through positive feedback loops. PI3K/AKT can activate NF-κB or other transcriptional regulators that indirectly increase HER2 expression, and ERK activation can enhance HER2 transcription by phosphorylating transcription factors, such as AP-2, which bind to the HER2 promoter and drive gene expression [19]. These processes further upregulate HER2 transcription, sustaining oncogenic signalling through a positive feedback loop. HER2 signalling can also crosstalk with the Wnt/β-catenin pathway; HER2 activation can stabilize β-catenin by inhibiting its degradation, leading to its nuclear translocation and transcriptional activation of Wnt target genes that drive proliferation, epithelial-to-mesenchymal transition (EMT), and tumour progression [23].

### 2.2. Mechanisms of HER2 Overexpression in EGA

The most common mechanism underlying HER2 overexpression is gene amplification, a genetic event where multiple copies of the ERBB2 gene lead to increased transcription and therefore increased levels of HER2 protein. Overexpression, by contrast, is a cellular state, where an increased level of HER2 protein is found on the cell surface, which may arise from amplification, but can also result from epigenetic modifications or altered post-transcriptional regulation, independent of gene copy number changes [24]. Less frequently, genetic mutations in ERBB2 can constitutively activate the receptor or its downstream signalling pathways, even without increased HER2 protein levels [25]. Such mutations, including tyrosine kinase domain alterations, which are common, may confer sensitivity to certain HER2-targeted tyrosine kinase inhibitors (e.g., Lapatinib), but they do not always predict the response to monoclonal antibody-based therapies like trastuzumab.

Clinically, assessment of HER2 status in EGA relies primarily on immunohistochemistry (IHC) and in situ hybridization (ISH) techniques. IHC detects the HER2 protein on a tumour’s cell surface, providing a measure of overexpression, while ISH, including fluorescence (FISH) and chromogenic (CISH) approaches, identifies gene amplification by quantifying the ERBB2 copy numbers relative to chromosome 17. Neither IHC nor ISH reliably detects the activating ERBB2 mutations, which may constitutively activate the receptor independently of HER2 protein abundance at the cell surface or gene copy number; identifying such mutations requires next-generation sequencing (NGS) or PCR-based molecular assays, which are becoming increasingly clinically relevant technologies for picking up on the extended genetic profiles of cancers as more targeted treatments are developed [26,27].

The current guidelines consistently recommend HER2 testing in advanced or metastatic EGA to guide targeted therapy. The joint ASCO/CAP/ASCP guidelines specify a two-step algorithm: IHC as the initial test, with ISH performed for IHC 2+ (equivocal) cases, using gastric-specific scoring criteria to account for incomplete and heterogeneous membrane staining. Adequate sampling is emphasized in the guidelines, with a minimum of five (ideally six–eight) biopsy fragments recommended to minimize sampling error, and laboratory quality assurance is emphasized [28,29]. The NCCN Gastric Cancer Guidelines adopt the same testing approach and recommend testing at the time of diagnosis of metastatic disease or relapse, with consideration of NGS to detect actionable ERBB2 mutations in selected patients [30]. The ESMO living guidelines, last updated in 2024, similarly recommends HER2 testing in all advanced/metastatic cases, recognizes the heterogeneous HER2 expression patterns in EGA tumours, and supports integrating NGS where targeted therapies are available.

### 2.3. Clinical Significance of HER2 in EGA

HER2 is overexpressed or amplified in ~20% of EGA [10]. HER2-positive cancers are thought to be invasive and associated with a poor prognosis [31]. Trastuzumab is a monoclonal antibody that targets the HER2 receptor and has been administered in the context of HER2-positive breast cancer patients for over 20 years, where it has been shown to improve progression-free survival (PFS) and OS [32,33]. However, treatment resistance over time can limit its utility as a first-line agent [34]. The reason for this resistance is likely due to acquired resistance mechanisms due to the interplay between a tumour and the tumour immune microenvironment [35]. The ToGA trial established the role of HER2 as a therapeutic target among patients with advanced and metastatic EGA, demonstrating a significant survival benefit among those who had HER2 overexpression treated with trastuzumab in addition to standard chemotherapy [12]. Since the ToGA trial, HER2-directed treatment has been considered the standard of care for patients with advanced HER2-positive EGA. Further advancements in HER2-directed treatment followed with the DESTINY-Gastric01 and DESTINY-Gastric02 trials, which further demonstrated that second-line T-Dxd, an antibody–drug conjugate (ADC) comprising an anti-HER2 antibody and a cytotoxic topoisomerase I inhibitor, improved OS among patients with advanced HER2-positive gastric and oesophagogastric junction adenocarcinoma [36,37]. In recent years, HER2 classification has moved from being a binary, positive or negative characterization, to including HER2-low and HER2-ultralow. These categories are better established in breast cancer, where clear definitions have been established [38]. Notably, the phase III DESTINY-Breast04 (DB-04) trial provided evidence that T-DXd significantly prolongs OS compared with standard chemotherapy in previously treated patients with HER2-low advanced breast cancer [39]. Although best characterized in a breast cancer setting, studies have also looked at HER2-low prevalence across other solid organ tumours, where it has been found to account for approximately 47% of breast cancers and at similar rates in gastric, salivary gland, lung, endometrial, and urothelial cancers [40]. An Italian study looked at the prevalence of HER2-low in EGA and found it ranged from 19.1% to 40.6% [41]. Understanding the impact of HER2 on the TME and resistance to treatment on what is now a spectrum of expression rather than a binary measure is increasingly important, as HER2-low is being explored and researched for its therapeutic targeting potential in EGA [36,41]. In breast cancers, HER2-low and -ultralow show distinct immune activation patterns, and understanding this interplay with the TME will be important for future trial design and therapeutic development [42].

The current international guidelines uniformly recommend HER2-directed therapy for patients with advanced or metastatic EGA who are HER2-positive. The NCCN Gastric Cancer Guidelines recommend the addition of trastuzumab to a fluoropyrimidine- and platinum-based chemotherapy backbone as the preferred first-line regimen, with HER2 testing required prior to treatment initiation [30]. Similarly, the ESMO guidelines recommend routine HER2 testing in all patients with metastatic disease and support trastuzumab in combination with first-line chemotherapy. Both the NCCN and ESMO guidelines, as well as recent ASCO expert consensus statements, recognize T-DXd as a second-line option for patients whose disease has progressed on trastuzumab-based therapy, reflecting the survival benefit demonstrated in the DESTINY-Gastric01 and DESTINY-Gastric02 trials [36,37,43].

## 3. The TME in EGA

The TME in EGA is a complex system comprising a cellular component made up of stromal cells, immune cells, and endothelial cells, and a non-cellular component made up of the extracellular matrix (ECM) and soluble mediators, such as cytokines and chemokines, that together influence tumour growth and progression [44,45]. The TME evolves with a tumour and supports cancer cell survival, local invasion, and metastatic dissemination [46]. The rapid proliferation of cancer cells creates a hypoxic and acidic environment, which activates pro-angiogenic pathways [45,47]. The resulting blood vessels are deficient and chaotic and perpetuate the hypoxic condition of the TME. The poor blood flow contributes to the poor differentiation of cancer cells, as well as the dysfunctional endothelial cells failing to produce the effective adhesion molecules that are required for immune cell extravasation to the cancer site [45].

Stromal cells, including cancer-associated fibroblasts (CAFs) and endothelial cells, contribute to extracellular matrix (ECM) remodelling, secretion of growth factors, and promotion of angiogenesis, creating a supportive niche for tumour growth [45]. The immune cells within the TME are highly heterogeneous, comprising cytotoxic T lymphocytes; regulatory T-cells; natural killer (NK) cells; macrophages; and immunosuppressive cells, such as CAFs, tumour-associated macrophages (TAMs), and myeloid-derived suppressor cells (MDSCs). These immune cells can exert either anti-tumour or pro-tumour effects depending on the context [46]. A state of immunosuppression prevails within the TME due to the increased expression of inhibitory immune checkpoint ligands on tumour cells and their corresponding receptors on immune cells. Engagement of these checkpoints with T-cells, NK cells, and antigen-presenting cells suppresses their anti-tumour activity and shifts them toward a regulatory, immunosuppressive phenotype, ultimately fostering tumour immune evasion and progression [45,48]. The ECM itself provides structural support and facilitates cell adhesion, migration, and signalling, while abnormal ECM composition and stiffness can promote invasion and resistance to therapy. CAFs are the main source of ECM, whereas MMPs are proteases that break down ECM proteins and are crucial in remodelling the ECM to promote tumour progression and metastasis [46]. Other key players in ECM remodelling include the cyclo-oxygenase (COX)-2–prostaglandin E(PGE)-2 axis and lysyl oxidases (LOXs) expressed and released by stromal cells. Furthermore, recent evidence suggests that YAP/TAZ, which act as mechanotransducers in response to ECM stiffness, may be upregulated in HER2+ EGA contributing to adaptive resistance through activation of downstream pathways and promoting immune evasion [49]. Stiffening of the ECM not only limits immune cell infiltration and activation, but also promotes metastasis by inducing epithelial–mesenchymal transition (EMT) through integrin-mediated activation of tumour cell survival and proliferation pathways.

The TME in EGA differs from that of other cancers in several ways that underscore the challenge of targeting this tumour type. Firstly, EGA exhibits marked intratumoural heterogeneity, which drives patchy immune infiltration and variable expression of targets, such as HER2 and PD-L1 [50]. Furthermore, the immune milieu is often “cold”, with a relatively low T-cell infiltration compared with melanoma or small-cell lung cancer, which may account for the relatively poor response to immunotherapy in comparison to other cancer types [48]. In addition, the EGA stroma is frequently fibrotic and desmoplastic, with a dense extracellular matrix (ECM) that impedes immune cell infiltration and potentially impacts drug penetration [51]. This fibrotic ECM and the resulting immune cell exclusion can drive the spatial heterogeneity of expression of markers, such as PD-L1, thereby creating immunologically “cold” areas that are resistant to an immune checkpoint blockade [52].

## 4. Modulation of the TME by HER2

**Figure 2:**
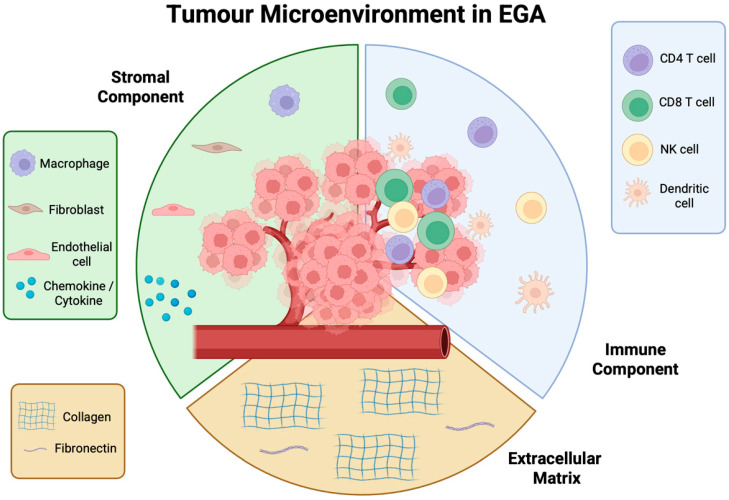
Tumour microenvironment (TME) in oesophagogastric adenocarcinoma (EGA). A complex ecosystem made up of a stromal component, extracellular matrix, and immune cell component. Each part of the TME plays a key role in the support and proliferation of cancer, which if left unchecked, will lead to dissemination and metastases.

The TME plays a crucial role in cancer progression, metastasis, and therapy resistance. HER2-positive tumours actively remodel and alter the TME to optimize tumour growth and progression. HER2 can impact all components of the TME, from the stromal cells, vasculature, ECM, and the immune landscape (Figure 2).

### 4.1. HER2 Impact on Stromal Cells

The tumour stroma consists of a variety of cell types that support cancer progression, such as CAFs, adipocytes, and MDSCs. CAFs drive tumour growth and facilitate immune evasion by remodelling the extracellular matrix, releasing growth factors, and exerting immunosuppressive effects [53]. In HER2-positive tumours, CAFs are activated via HER2-driven pathways, such as PI3K/AKT and mTOR. Once activated, CAFs release growth factors, including fibroblast growth factor (FGFs), hepatocyte growth factor (HGF), and insulin-like growth factor (IGF) that promote tumour cell proliferation, motility, and invasion [54]. They also secrete chemokines like CXCL12, which establish chemotactic gradients that facilitate cancer cell migration within the TME [54].

CAFs are also heavily involved in ECM remodelling. HER2-activated CAFs enhance the synthesis of ECM components, such as fibronectin, collagen, and biglycan, leading to greater ECM stiffness and density, which in turn creates a mechanical barrier which can impact the infiltration of immune cells and also the penetration of therapeutic agents [54]. Fibrosis-associated mediators like connective tissue growth factor (CTGF) and lysyl oxidase (LOX) are also released, which drives collagen cross-linking and ECM remodelling [55]. These alterations reinforce the immunosuppressive TME by limiting immune cell penetration and skewing macrophages toward a pro-tumorigenic M2 phenotype [56,57].

The secretory cytokine TGF-β is a crucial mediator of tumour–stroma crosstalk in the TME [58]. Compared to other fibroblasts, CAFs have more proliferative potential, which is stimulated by the TGF-β present in the TME [58]. TGF-β also promotes EMT, enhancing cancer cell motility, invasiveness, and stemness [54]. ECM remodelling further supports EMT by providing mechanical cues that drive cytoskeletal changes. This fosters cancer stem cell (CSC) formation, which thrives in a CAF-modulated microenvironment. CSCs and CAFs engage in a reciprocal feedback loop, reinforcing a tumour-promoting niche that drives progression and immune evasion [54].

### 4.2. HER2 Impact on Angiogenesis

Angiogenesis is a hallmark of cancer and is driven by a tumour’s requirement for oxygen and nutrients to feed its rapid growth [59]. HER2-positive tumours show increased angiogenesis due to HER2-mediated activation of the PI3K/AKT and mTOR pathways [19]. This signalling upregulates hypoxia-inducible factors (HIF-α), which in turn promote VEGF secretion, a major mediator of angiogenesis [60]. HER2-mediated signalling can also upregulate COX-2, which drives PGE2 production and activates HIF-1α and NF-κB, further enhancing VEGF expression [54]. Additionally, HER2-positive cancer cells can undergo vasculogenic mimicry by overexpressing VE-cadherin, forming vessel-like structures that support tumour survival under hypoxic conditions [54,61]. Despite the process of angiogenesis, the vessels that result are often leaky and abnormal and contribute to a hypoxic environment, which in turn further drives HIF-1 α and VEGF into a negative feedback loop [54]. These defective and leaky vessels can contribute to inefficient drug delivery to the tumour bed, impacting efficacy of chemotherapy agents [62].

### 4.3. HER2 Impact on Immune Cells

HER2 signalling actively remodels the TME in EGA by dampening antigen presentation pathways and promoting recruitment of immunosuppressive cell populations, including TAMs and Tregs. Through these mechanisms, HER2 amplification facilitates immune evasion and creates a microenvironment less responsive to anti-tumour immunity and immunotherapy [16]. A recent study looked at the difference in immune cell infiltrates across HER2-positive, HER2-negatve, and HER2-low tumours in EGA, and found that HER2-low tumours had significantly greater immune cell infiltration compared with HER2-positive tumours, suggesting a more inflamed and immunogenic tumour immune microenvironment (TIME) [63]. They also showed that immune-oncology (IO)-related gene expression, including PD-1/PD-L1 and IFN-γ signatures, was lowest in HER2-positive tumours, suggesting an intrinsically less immunogenic TIME [63]. In the TRAP cohort study of 83 oesophageal adenocarcinoma biopsies, the HER2-positive tumours were enriched for epithelial markers (EPCAM, E-cadherin), but exhibited lower immune cell infiltration (CD8+ T-cells, NK cells) and reduced expression of immune exhaustion markers (PDCD1LG2, CTLA4). The non-responders to anti-HER2 therapy showed increased baseline immune exhaustion, hypoxia, and VEGF signalling, suggesting that resistance may involve an immune-suppressive and pro-angiogenic TIME, and that dual HER2-VEGF targeting could be a future strategy [35]. These studies indicate that HER2-positive EGA has a relatively “immune-cold” TIME, with fewer cytotoxic lymphocytes and lower immune activation, which may limit checkpoint inhibitor efficacy. This supports combining HER2-targeted therapy with immunotherapy and exploring VEGF inhibition to overcome resistance [35,45]. This concept of “immune-hot and cold” is not new and has been established as a stratification method to guide targeted treatment of colorectal cancer (CRC). This followed an observation that the composition, abundance, and spatial distribution of immune cells within a tumour could predict survival in CRC more accurately than the traditional TNM staging system [64,65]. This led to the development of the Immunoscore in CRC, which is a standardized, validated scoring system that is based on the quantification of CD3 and CD8 lymphocytes at two locations: the tumour centre and the margin [66,67]. The score represents the first time that tumours have been classified according to immune infiltration rather than tumour histology. The consensus Immunoscore has since been globally validated in colon cancer and shown to outperform traditional prognostic markers, such as the pT stage, pN stage, lymphovascular invasion, tumour differentiation, and MSI status [66,68]. Whilst no equivalent immune-based classification is yet established for EGA, efforts are underway to stratify it according to the immune cell subtypes and gene signatures within the TME [69]. The aforementioned studies evaluating immune cell infiltration, PD-L1 expression, and TIME phenotypes in EGA suggest that a similar framework could refine prognostication and guide patient selection for immunotherapy in the future, with risk stratification taking the HER2 status into consideration. The TME score is an emerging prognostic biomarker that can help predict efficacy of ICIs [70].

The immunosuppressive TME of HER2-positive tumours is characterized by low infiltration of cytotoxic CD8+ T-cells and NK cells [35]. Trastuzumab mediates tumour killing both by inhibiting HER2 signalling and through antibody-dependent cellular cytotoxicity (ADCC), which recruits NK cells and cytotoxic T-cells. A tumour’s upregulation of PD-L1/PD-L2, driven by HER2 signalling, cytokines, or hypoxia, suppresses these effector cells and limits ADCC, reducing therapeutic efficacy. This forms the rationale for combing HER2-targeted therapy with PD-1/PD-L1 inhibitors, such as pembrolizumab, which can restore immune activity and enhance ADCC.

### 4.4. TME Implications for HER2-Targeted Treatment

A study looked at the remodelling that occurs within the TIME in response to HER2-directed treatment with trastuzumab in advanced gastric cancer, and found that there was a significant increase in the infiltration of NK cells and CD8 T-cells indicating an enhanced, “immune-hot” response following HER2-targeted treatment [71]. Another study observed that preoperative chemotherapy combined with trastuzumab increased CD8+ T-cell infiltration and reduced the FoxP3+ T-cell presence compared with chemotherapy alone in HER2-positive gastric cancer, suggesting that trastuzumab may help establish a more activated, anti-tumour TIME [72].

A recent study assessing the impact of the TIME on the efficacy of T-Dxd in gastric cancer found that T-DXd treatment increased tumour-infiltrating CD8+ and PD1+CD8+ T-cells and upregulated cytotoxic and helper T-cell gene signatures, while downregulating hypoxia, MYC, collagen, and IL-10 pathways, suggesting that both the baseline HER2 levels and the TIME influence therapeutic efficacy, and that T-DXd may actively modulate the TIME [73].

It is important to acknowledge what a difference in HER2 status means across different tumour types when considering the impact of HER2-targeted treatments. HER2 positivity in breast cancer can promote T-cell and monocyte recruitment in trastuzumab-sensitive tumours [16], whereas the “immune-cold” phenotype in HER2-positive EGA, with lower CD8+ T-cell and NK cell infiltration compared with HER2-negative tumours, contrasts with this. The relationship between HER2 status and immune infiltration is tumour-dependent and may influence the response to HER2-targeted therapies differently across cancers, which should be considered in future trial development.

## 5. Therapeutic Resistance to HER2-Targeted Treatment

**Figure 3:**
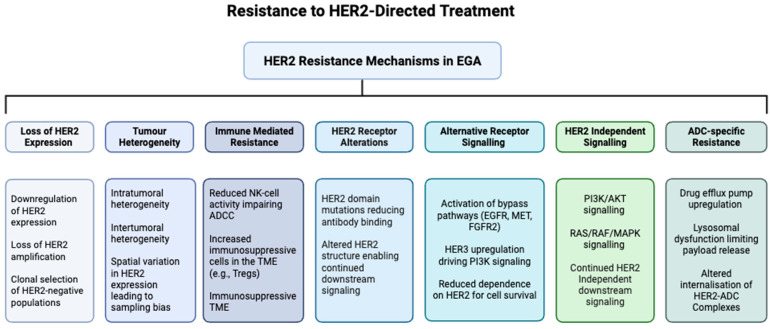
Resistance mechanisms to HER2-directed treatment of EGA. HER2-directed therapies for EGA face multiple mechanisms of resistance, including loss or reduction in HER2 expression due to clonal selection; structural alterations of the HER2 receptor, such as mutations that impair drug binding; upregulation of alternative receptor tyrosine kinases, including EGFR, MET, FGFR2, and HER3; constitutive activation of downstream signalling pathways, such as PI3K/AKT and RAS/RAF/MEK/ERK; intratumoural (with mixed HER2-positive and HER2-negative clones) and intertumoural heterogeneity (between primary and metastatic sites) reducing therapeutic efficacy; immune-mediated resistance through an impaired ADCC and an immunosuppressive TME; and ADC-specific mechanisms, including drug efflux, lysosomal dysfunction, altered trafficking, and payload resistance, collectively limiting the effectiveness of current HER2-targeted therapies.

Although targeted therapy against advanced HER2-positive EGA has delivered clinical benefits, many patients eventually experience disease progression as resistance emerges. There are multiple mechanisms that may underlie this therapeutic resistance (Figure 3).

### 5.1. HER2 Heterogeneity

Most notably, EGA is characterized by marked intra- and intertumoural heterogeneity, a feature strongly linked to its poor prognosis [74]. HER2 heterogeneity in EGA encompasses variations in the expression status and gene copy number, both of which can influence treatment outcomes. Alterations, such as HER2 mutation, downregulation, or modification may affect therapeutic efficacy, with evidence suggesting that higher HER2 amplification correlates with improved survival following trastuzumab therapy [75,76]. In HER2-positive EGA, intratumoural heterogeneity denotes the spatial coexistence of distinct tumour subclones at baseline, while temporal heterogeneity post-therapy captures the adaptive evolution of tumour cells and the microenvironment in response to therapeutic pressure. Previous studies have shown that HER2 positivity is frequently lost after first-line therapy, likely secondary to clonal selection during exposure to trastuzumab [77]. The same study, interestingly, found a trend towards better PFS and OS in patients with longer trastuzumab-free intervals, suggesting a benefit in the timing of a “wash-out” period [77].

### 5.2. Impaired Receptor Function and Binding

Mutations in the HER2 gene are another recognized mechanism of resistance to anti-HER2 therapy, as they can alter the receptor structure and impair trastuzumab binding, resulting in treatment failure. Such mutations are particularly associated with innate resistance rather than acquired resistance [74]. Specific HER2 gene fusions, such as SNF270-HER2, and point mutations such as V777L and S310F, can prevent binding of agents like Lapatinib and Pertuzumab [78,79]. Another method of resistance by impaired HER2 receptor binding has been linked to signal transducer and activator of transcription 3 (STAT3) hyperactivation, which drives overexpression of MUC1 and MUC4, which are mucins that can block trastuzumab binding and sustain HER2 signalling, and represent emerging biomarkers of poor trastuzumab response [78]. Similarly, overexpression of truncated DARPP-32 prevents receptor dephosphorylation, further contributing to treatment resistance [78].

### 5.3. Activation of Alternative Signalling Pathways

Downstream signalling pathways are also implicated in HER2-directed treatment resistance in EGA. The evidence suggests that activating mutations in PI3K can drive primary resistance to HER2-targeted therapies [74], whereas the activation or inhibition of the PI3K/AKT and MAPK pathways can result in acquired resistance [80]. The upregulation and activation of Src family kinases, including Src and YES1, contribute to acquired trastuzumab resistance in HER2-positive gastric cancer by sustaining MAPK/ERK and PI3K/mTOR signalling. Furthermore, loss of tumour suppressors, such as PTEN, enhances PI3K/AKT signalling, reducing sensitivity to trastuzumab and other HER2-directed agents [74].

### 5.4. Contributions of the TME to Resistance

#### 5.4.1. Hypoxia and Angiogenesis

Aberrant angiogenesis in the TME of HER2-positive EGA creates regions of hypoxia, which activate hypoxia-inducible factor (HIF) pathways and upregulate survival and pro-angiogenic signals, such as VEGF. Hypoxia also promotes immunosuppression by inhibiting T- and NK cell activity, limiting ADCC, and driving the epithelial-to-mesenchymal transition (EMT), enhancing tumour invasiveness. Together, these factors reduce the efficacy of HER2-targeted therapies and contribute to both primary and acquired treatment resistance.

#### 5.4.2. EMT Pathway

The EMT represents a key adaptive mechanism by which epithelial tumour cells acquire mesenchymal traits, which enables tumour cells to acquire a more migratory, invasive phenotype and resist apoptosis, and is thought to play a key role in drug resistance [59,78]. Trastuzumab-resistant cancer cells exhibit an EMT-like phenotype by promoting the Wnt signalling pathway [81]. EMT can also follow from increased dimerization of HER2 with HER4 and subsequent binding of yes-associated protein 1 (YAP1) [82].

## 6. Emerging HER2 Therapeutic Strategies

### 6.1. Antibody Drug Conjugates (ADCs)

ADCs targeting HER2 have emerged as promising therapies for advanced EGA. T-Dxd combines trastuzumab with a topoisomerase I inhibitor via a cleavable linker, inducing cytotoxicity by disrupting DNA replication and transcription. Phase II trials (DESTINY-Gastric01/02) have demonstrated improved response rates and OS, leading to its FDA approval as a second-line treatment for advanced HER2-positive EGA in 2021 [36,37]. Its use has been expanded into a first-line setting following DESTINY Gastric-03, which showed that T-DXd, in combination with fluoropyrimidine (FP) chemotherapy and pembrolizumab in a first-line setting, demonstrated promising anti-tumour activity and manageable safety, particularly at a lower T-DXd dose of 5.4 mg/kg [83]. DESTINY Gastric-04, an international phase III trial, most recently directly compared second-line T-DXd with ramucirumab plus paclitaxel, demonstrating a significantly longer OS (14.7 vs. 11.4 months) and higher objective response rates (ORR) (44.3% vs. 29.1%) with T-DXd, confirming its superiority over standard therapy [84]. The use of T-Dxd in pre-treated HER2-low EGA has also been investigated, with recent preliminary evidence showing that T-DXd has clinical activity in this patient cohort, albeit with a small patient sample size of 45 [85]. Similar to the acquired resistance patterns seen with trastuzumb treatment, resistance has also been observed with T-Xd and can limit its efficacy. Given the more complex structure of ADCs and the multifaceted mechanisms of action, resistance can arise at any point in the process. It may stem from altered antigen expression or recognition, impaired drug internalization or degradation, or disrupted payload release [86]. The TME itself may also play a part in acquired resistance to T-DXd by creating structural barriers through a dense ECM that restricts the ADC penetration and prevents uniform distribution within a tumour [87]. Efforts to overcome resistance to ADCs include the development of bispecific-targeting ADCs. By engaging multiple tumour-associated antigens at once, bispecific agents can theoretically improve the targeting precision and payload internalization, while helping to overcome the resistance seen with current ADCs that is due to alternate signalling pathways [87]. Other approaches targeting acquired resistance include combination therapy to increase efficacy.

### 6.2. Combination HER2/Immune Checkpoint Inhibitors

As outlined herein, the combination of targeting HER2 and PDL1 blockades has an excellent mechanistic rationale, which has been firmly corroborated clinically following the landmark phase 3, randomized controlled clinical trial KEYNOTE-811, which showed that pembrolizumab combined with trastuzumab and chemotherapy significantly improved the OS compared to placebo plus trastuzumab and chemotherapy in patients with unresectable HER2-positive metastatic EGA, with a greater benefit observed in patients with a PD-L1 combined positive score (CPS) ≥ 1 [14]. From the interim analysis, the PFS was improved (10.0 vs. 8.1 months; HR 0.72; *p* = 0.0002), and later the final analysis of overall survival demonstrated a significant OS benefit (20.0 vs. 16.8 months; HR 0.80; *p* = 0.0040). The results of both the interim analysis assessing the PFS and objective response rates and the final analysis of OS presented at ESMO 2024 supported the approval and establishment of this regimen as the first-line standard of care for this patient cohort [13,14]. Although the OS was significantly improved, the modest 3.2-month gain raises questions about the balance between benefit, toxicity, and cost compared with the previous standard treatment. The immunomodulatory impact of trastuzumab in HER2-positive EGA may well help to explain the positive results seen in KEYNOTE-811 by priming the TIME toward a more activated state; trastuzumab may increase responsiveness to an immune checkpoint blockade [13,14]. The exploration of combination treatments based on patient biomarker status is an exciting and rapidly evolving landscape in precision oncology. Further questions that stem from KEYNOTE-811 include what the best approach for PDL1-negative tumours will be, whether or not chemotherapy can be de-escalated whilst maintaining survival efficacy, and the best approach for HER2-directed treatment with the expansion of ADCs, such as T-DXd, in the oncology landscape. The later question will be addressed by the DESTINY-Gastric 05 trial, which is currently recruiting and will compare first-line T-DXd plus pembrolizumab + chemotherapy versus trastuzumab + pembrolizumab + chemotherapy in HER2-positive, PD-L1 CPS ≥ 1 gastric/junctional cancer, with an exploratory cohort testing T-DXd-based therapy in CPS < 1 disease (NCT06731478) [88].

### 6.3. Bispecific Antibodies

Bispecific antibodies are engineered molecules that bind two distinct antigens or epitopes simultaneously, enabling a more comprehensive blockade of signalling pathways and enhanced anti-tumour immunity. For HER2/HER2 bispecifics, such as Zanidatamab, dual-epitope engagement promotes more efficient HER2 receptor internalization and degradation than is achieved with trastuzumab alone, leading to better pathway suppression within the TME [89]. In a phase II study of first-line HER2-positive advanced EGA, Zanidatamab plus chemotherapy achieved a confirmed objective response rate (cORR) of 76%, with a median duration of response of 18 months; the median PFS and OS were 12 and 36 months, respectively, with a manageable safety profile [90]. The ongoing phase III HERIZON-GEA-01 trial is comparing Zanidatamab + chemotherapy (+/−Tislelizumab) with standard trastuzumab-based therapy and the results are awaited [91]. For HER2/PD-1 or HER2/PD-L1 bispecific formats, co-localizing checkpoint inhibition at the HER2-expressing tumour sites may further enhance recruitment of cytotoxic T-cells and NK cells (as described in Section 4.3). In turn, this could lead to greater T-cell activation and counteract HER2-driven immune exclusion, offering a rationale for combining HER2 targeting with immune checkpoint blockade.

### 6.4. Dual HER2 Blockade

A recent phase II feasibility study demonstrated that a dual HER2 blockade with trastuzumab and Pertuzumab in a curative setting for oesophageal cancer was well tolerated in addition to neoadjuvant chemoradiation, with better R0 resection rates in the treated group, calling for phase III trials to assess the survival outcomes [92]. However, in a metastatic setting, the phase III JACOB trial investigated the addition of Pertuzumab to trastuzumab and chemotherapy, and did not significantly improve the OS in patients with HER2-positive metastatic EGA compared with placebo, thereby questioning the clinical value of a dual HER2 blockade in HER2-positive EGA [93]. The phase II INNOVATION trial evaluated perioperative chemotherapy (CT) alone or combined with trastuzumab (T) or trastuzumab plus Pertuzumab (T + P) in 172 patients with resectable HER2-positive EGA. After a protocol amendment to incorporate FLOT-based CT, the major pathological response rates improved across all arms, particularly with CT + T with FLOT as the backbone. However, after a median follow-up of 4.3 years, the PFS and OS showed only non-significant improvements with CT + T compared to CT alone, while CT + T + P appeared detrimental. These trials highlight the challenges of using the pathological response as a surrogate endpoint [94].

### 6.5. Combination Approaches Targeting HER2 and the TME

#### 6.5.1. Tyrosine Kinase Inhibition

Lapatinib, a tyrosine kinase inhibitor (TKI) targeting HER2 and EGFR, blocks downstream signalling by inhibiting tyrosine phosphorylation, suppressing tumour growth, and inducing apoptosis. However, in clinical trials such as LoGic and TyTAN, lapatinib failed to show benefit in HER2-positive EGA [95,96].

#### 6.5.2. Anti-Angiogenic Agents

Targeting angiogenesis has been a central approach in cancer treatment in recent decades, with multiple anti-angiogenic agents having gained FDA approval and now integrated into standard clinical practice. Anti-angiogenic therapy is constrained by class-related toxicities (hypertension, bleeding, thromboembolism, and proteinuria) and a lack of validated predictive biomarkers, limiting precision in patient selection [97,98]. Bevacizumab is a monoclonal antibody that targets circulating VEGF-A, blocking its interaction with VEGF receptors. It is approved for use in combination with fluorouracil-based chemotherapy as a first- and second-line therapy for metastatic colorectal cancer (mCRC).

Ramucirumab, a monoclonal antibody targeting the VEGFR-2 extracellular domain, is approved as a second-line treatment for advanced or metastatic EGA, either as a monotherapy or combined with paclitaxel. This approval followed double-blind, placebo-controlled studies, which demonstrated improved OS with ramucirumab [99]. The phase III RAINBOW trial confirmed these findings, showing a median OS of 9.6 months with ramucirumab plus paclitaxel versus 7.4 months with placebo plus paclitaxel, establishing the combination as a standard second-line therapy [100].

Trastuzumab combined with ramucirumab and paclitaxel showed promising activity in second-line HER2-positive EGA, with an objective response rate of 54% and manageable toxicity reported. However, loss of HER2 expression after first-line therapy was observed in 34.8%, highlighting a problem with acquired resistance with this approach [101].

## 7. Future Directions

This review highlights the dynamic interplay between HER2 signalling, tumour heterogeneity, and the TME in EGA. Future research should focus on optimizing HER2-targeted therapies with immunomodulatory approaches, including checkpoint inhibitors, bispecific antibodies, and ADCs, to overcome both primary and acquired resistance. Stratifying patients by HER2 expression across a spectrum (high, low, or ultralow rather than binary measures), immune infiltration patterns, and TME characteristics may enable more personalized therapeutic strategies. The issue of intratumoural heterogeneity may be addressed in the future by digital pathology analysis, which can provide quantitative, whole-slide assessment of HER2 expression, enabling detection of focal positive clones and spatial patterns that may be missed by conventional scoring. Ongoing trials of HER2-directed therapy, particularly in the era of ADCs, in curative settings also raise the possibility of expanding the clinical benefit beyond advanced and metastatic disease in the future. Future trial designs may also incorporate dynamic changes in HER2 expression overtime, adjusting treatment approaches accordingly. Furthermore, emerging technologies, such as single-cell RNA sequencing and spatial transcriptomics, which can enable high-resolution mapping of TME-HER2 interactions, revealing patterns of cell interaction, spatial immune exclusion, and heterogeneity in HER2 expression, may refine therapeutic stratification in the future [102,103].

## 8. Conclusions

While HER2-targeted therapies have improved outcomes for EGA, the TME plays a pivotal role in modulating the treatment response and driving resistance. Immunosuppressive conditions, hypoxia, and aberrant downstream signalling within the TME can limit the efficacy of HER2-directed agents. Emerging strategies, such as ADCs, bispecific antibodies, and combination immunotherapies, aim to simultaneously target HER2 (across the spectrum of expression) and remodel the TME, offering the potential for more durable and effective therapies for EGA. Looking ahead, the integration of HER2 and TME biomarkers, including spatial, cellular, and immunologic features into prospective clinical trial designs, will be essential for achieving true precision therapy selection in EGA.

## Data Availability

No new data were created or analysed in this study. Data sharing is not applicable to this article.
